# Scleral ulceration after vitreoretinal surgery

**DOI:** 10.4103/0301-4738.53059

**Published:** 2009

**Authors:** Nikhil S Gokhale

**Affiliations:** Gokhale Eye Hospital and Eyebank, Anant Building, Gokhale Road (S), Dadar (West), Mumbai - 400 028, India

**Keywords:** Conjunctival flap, scleral ulcer, vitreoretinal surgery

## Abstract

Scleral ulceration after ocular surgery is a rare but serious complication. Determination of the underlying systemic and local causes is critical for treatment. An unusual case of ischemic scleral ulceration after vitreoretinal surgery in a diabetic patient is reported. Patient was successfully treated with a pedicle conjunctival graft.

Scleral ulceration after ocular surgery can have diverse etiologies. Clinical workup and investigations can guide us to the possible etiology, so that we can treat it correctly. While immunosuppression is the treatment of choice in surgically-induced necrotizing scleritis, it may be disastrous in infectious ulceration and of no benefit in ischemic ulceration. A systematic approach in such rare clinical situations is discussed with the help of this case report.

## Case Report

A 40-year-old male patient was referred for scleral ulceration following vitreoretinal surgery. He had a previous history of laser photocoagulation for proliferative diabetic retinopathy in both eyes. Patient had a well-controlled insulin-dependent diabetes mellitus. He had a previous history of a diabetic foot problem. No other known systemic problems were present.

The patient had undergone right eye vitrectomy with silicone oil injection for a complicated diabetic retinal detachment. No scleral buckling was done. His postoperative course was uneventful in the first two weeks. On his follow-up visit at three weeks, scleral ulceration was noted and he was referred for further management.

**Figure 1:**
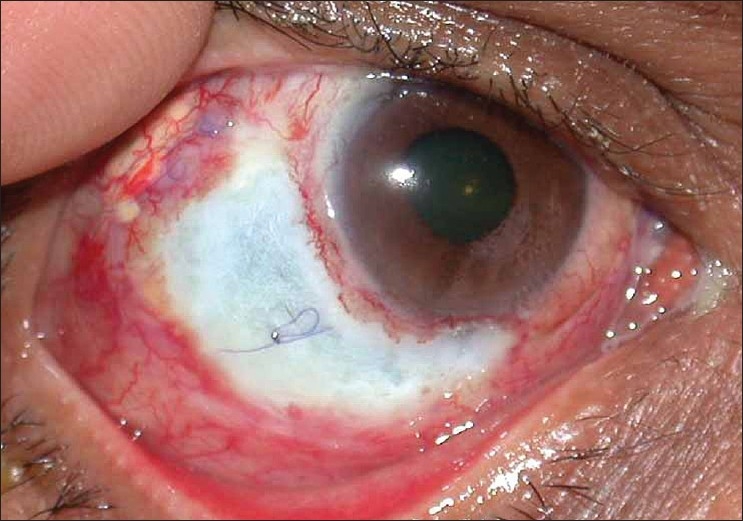
Preoperative picture showing scleral ulceration with exposed sclerotomy

On presentation, right eye had a vision of finger counting at one meter with accurate projection. Lids were normal, cornea was clear with few punctate erosions and a wide area of scleral ulceration was noted from 5 to 10'O clock position around the limbus [Fig. 1]. The base of the ulcer showed grey-white scleral necrosis with ischemic base but with no uveal show or purulent discharge. Sclerotomy site with a vicryl suture was visible. The conjunctiva over the ulcer was ulcerated and absent. The area around the ulcer showed conjunctival congestion but there was no evidence of scleral inflammation (deep congestion, with scleral thickening and edema in a diffuse or nodular pattern), or nodule formation. Anterior chamber was quiet, pupil was round and lens was clear. Fundus evaluation showed attached retina.

The patient was started on oral doxycycline 100 mg/day, carboxymethylcellulose drops 0.5% hourly and moxifloxacin 0.5% eye drops four times a day. Scrapings from the ulcer bed were subjected to smears and cultures but did not reveal any organisms. Gram's, KOH, calcoflour and acid-fast staining were used for evaluation of smears. Blood agar, chocolate agar, MacConkey's agar, brain heart infusion medium, Sabaroud's dextrose agar without cycloheximide were directly inoculated in the clinic and sent to the microbiology laboratory.

Rheumatoid factor, antinuclear antibody, anti-neutrophilic cytoplasmic antibody, complete blood counts with Erythrocyte sedimentation rate (ESR), C-reactive protein, Chest X-ray, blood sugars, liver function tests and serum creatinine were done under the guidance of a rheumatologist. These were done to detect collagen vascular disease and as baseline tests in case, systemic immunosuppression was deemed necessary. Clinical evaluation and laboratory tests for collagen vascular disease were negative.

**Figure 2:**
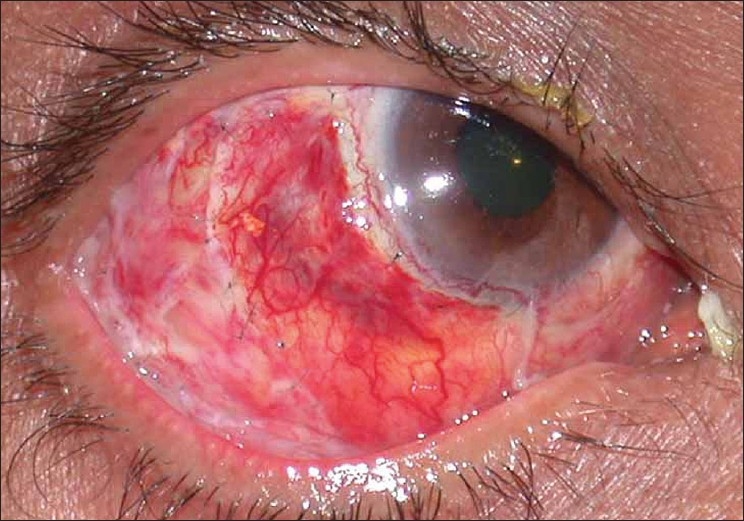
Postoperative picture showing conjuctival flap and amniotic membrane in place

**Figure 3:**
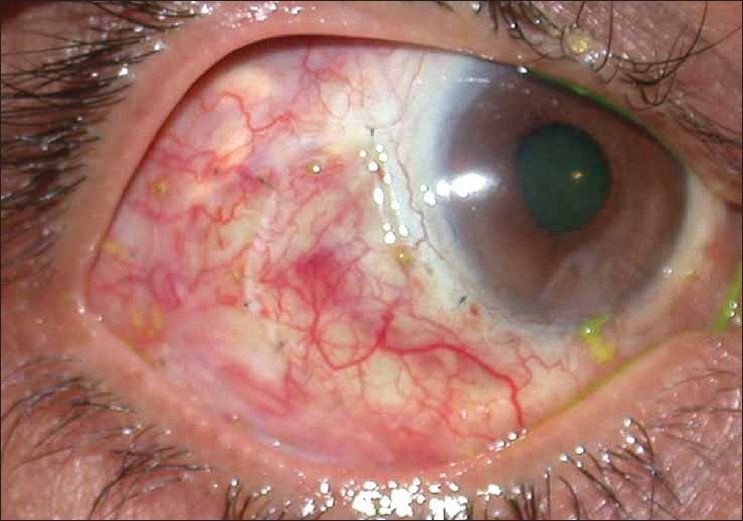
Two weeks postoperative picture

**Figure 4:**
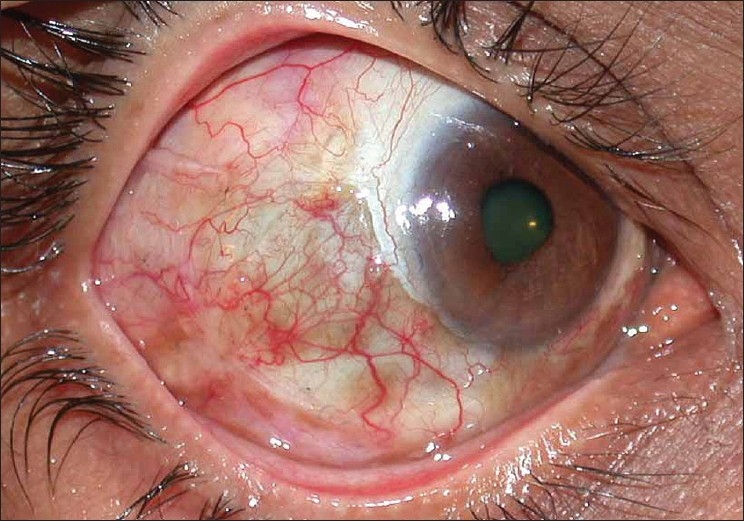
Two months postoperative picture

In view of the large area of ischemic ulceration, a rotational pedicle graft was considered so as to cover the bare area and also provide vascular supply. A large pedicle conjuctival flap was dissected from the uninvolved adjacent inferotemporal conjunctiva and was rotated anteriorly to cover the defect. It was sutured with multiple interrupted 10'0 nylon sutures. The large bare area created was then draped with a cryopreserved amniotic membrane and sutured similarly. Postoperatively moxifloxacin 0.5% drops were replaced with a combination of gatifloxacin 0.3% with dexamethasone 0.1% drops four times a day. Lubricants and oral doxycycline were continued. The graft was well taken up [Fig. 2] and the donor area also healed well. After two weeks [Fig. 3], antibiotic steroid drops were discontinued and a tapering dose of fluoromethalone eye drops were prescribed for a period of three weeks. Loose sutures were removed at one month and again at two months postoperatively [Fig. 4]. The patient did not show any recurrence or inflammation until one year of follow-up.

## Discussion

Scleral necrosis and ulceration after posterior segment surgery is rare and can have a varied etiology. Surgically-induced necrotizing scleritis[1] (SINS), local infection and ischemia due to overcauterization are the most likely causes. Conjunctival necrosis has been described following intravitreal triamcinolone[2] and subconjunctival injection[3,4] of triamcinolone and methylprednisolone. Scleral buckle infection[5] may also cause scleral inflammation and abscess formation. Mitomycin C[6] and irradiation[7] are known to cause scleral necrosis after use in pterygium surgery. They are not used in posterior segment surgery but a history of previous exposure to these may be contributory.

SINS following pars plana vitrectomy is rare and requires prompt and aggressive immunosuppression.[1] Differentiation from an infective postoperative process is essential since use of immunosuppressives in an infection would be detrimental. Excessive cauterization around the sclerotomy site could be another possible cause of scleral necrosis. Ischemic scleral necrosis due to overcauterization has been reported after bare sclera pterygium surgery.[8] This etiology would also not benefit from immunosuppression.

The case presented here did not have clinical evidence of active scleral inflammation around the area of ulceration, had clinically and serologically no evidence of collagen disease and recovered without any systemic immunosuppression. There was no clinical or laboratory evidence of an active infective process causing the scleral ulceration. No depot steroids were used and there was no previous exposure to antimetabolites and irradiation. The only likely possible cause was ischemic necrosis possibly due to overcauterization of the bed around the sclerotomy site. The patient improved after covering the ulcerated area with a pedicle conjunctival graft, which not only covered the bare surface but also provided blood supply through the pedicle.
